# Molecular characterization of *Glaesserella parasuis* strains isolated from North America, Europe and Asia by serotyping PCR and LS-PCR

**DOI:** 10.1186/s13567-021-00935-9

**Published:** 2021-05-12

**Authors:** Nubia Macedo, Marcelo Gottschalk, Katrin Strutzberg-Minder, Chao Nguyen Van, Lijun Zhang, Geng Zou, Rui Zhou, Thaire Marostica, Maria Jose Clavijo, Alexander Tucker, Virginia Aragon

**Affiliations:** 1 Veterinary Diagnostic Laboratory, Iowa State University, Ames, IA USA; 2 Faculty of Veterinary Medicine, University of Montreal, Montreal, Canada; 3 Innovative Veterinary Diagnostics (IVD GmbH), Albert-Einstein-Str. 5, 30926 Seelze, Germany; 4 Faculty of Animal Science and Veterinary Medicine, University of Agricultural and Forestry, Hue University, Hue, 53000 Vietnam; 5 State Key Laboratory of Agricultural Microbiology, Huazhong Agricultural University College of Veterinary Medicine, Wuhan, 430070 China; 6 Department of Veterinary Clinic and Surgery, Federal University of Minas Gerais, Av. Antônio Carlos, 6627, Belo Horizonte, MG Brazil; 7 PIC North America, Hendersonville, TN USA; 8 Department of Veterinary Medicine, University of Cambridge, Madingley Road, Cambridge, CB3 OES UK; 9 IRTA, Centre de Recerca en Sanitat Animal (CReSA, IRTA-UAB), Campus de la Universitat Autònoma de Barcelona, 08193 Bellaterra, Spain; 10 OIE Collaborating Centre for the Research and Control of Emerging and Re-Emerging Swine Diseases in Europe (IRTA-CReSA), Bellaterra, Barcelona, Spain

**Keywords:** *Glaesserella parasuis*, Serotyping, VtaA marker, LS-PCR

## Abstract

*Glaesserella parasuis* strains were characterized by serotyping PCR, *vtaA* virulence marker Leader Sequence (LS)-PCR, clinical significance, and geographic region. Overall, the serovars 4, 5/12, 7, 1, and 13 were the most commonly detected. Serovars of greatest clinical relevance were systemic isolates that had a higher probability of being serovar 5/12, 13, or 7. In comparison, pulmonary isolates had a higher likelihood of being serovars 2, 4, 7, or 14. Serovars 5/12 and 13 have previously been considered disease-associated, but this study agrees with other recent studies showing that serovar 7 is indeed associated with systemic *G. parasuis* disease. Serovar 4 strains illustrated how isolates can have varying degrees of virulence and be obtained from pulmonary, systemic, or nasal sites. Serovars 8, 9, 15, and 10 were predominantly obtained from nasal samples, which indicates a limited clinical significance of these serovars. Additionally, most internal *G. parasuis* isolates were classified as virulent by LS-PCR and were disease-associated isolates, including serovars 1, 2, 4, 5/12, 7, 13, and 14. Isolates from the nasal cavity, including serovars 6, 9, 10, 11, and 15, were classified as non-virulent by LS-PCR. In conclusion, the distribution of *G. parasuis* serovars remains constant, with few serovars representing most of the strains isolated from affected pigs. Moreover, it was confirmed that the LS-PCR can be used for *G. parasuis* virulence prediction of field strains worldwide.

**Supplementary Information:**

The online version contains supplementary material available at 10.1186/s13567-021-00935-9.

## Introduction

*Glaesserella parasuis* is an important pathogen of swine and remains one of the most important bacterial causes of mortality in swine production worldwide. Clinical signs of infected pigs range from fever, respiratory signs, and swollen joints with lameness, to central nervous signs, and sudden death. Chronic infections are also associated with reduced growth rates and food conversion efficiencies. The main pathological findings include fibrinopurulent polyserositis, polyarthritis, and meningitis, commonly known as Glässer’s disease [1].

Glässer’s disease is considered one of the most prevalent swine bacterial infections worldwide [2]. Reliance on prophylactic or strategic medication to control this disease is an ongoing concern regarding the emergence of antimicrobial resistance [3–6]. Bacterins and a live attenuated vaccine are commercially available. However, *G. parasuis* is a highly diverse pathogen, with still poorly known cross-protection against disease-causing field strains [7, 8]. In many countries, autogenous bacterins are also produced.

*G. parasuis* is also a commensal of the upper respiratory tract (URT) [9]. Disease outbreaks are associated with the introduction of novel *G. parasuis* strains into an immunologically naïve pig population, typically through the mixing of pigs from different sources. Other predisposing factors include poor management, high stocking density, inadequate ventilation, and coinfections with other respiratory pathogens [10, 11].

Epidemiological investigation of *G. parasuis*-associated disease, and preventive health management, relies on identifying specific *G. parasuis* serovars colonizing and or causing illness. Fifteen *G. parasuis* serovars that differ in virulence have been described [12, 13]. However, healthy pigs can carry serovars commonly isolated from systemic sites in the URT, alongside isolates of serovars rarely identified in clinical disease [14, 15].

This broad diversity within the *G. parasuis* species in the field requires surveillance tools to detect disease-relevant strains. Whole-genome sequencing enabled detailed analysis of the capsule loci of *G. parasuis* [16] and the development of a multiplex serotyping PCR [17, 18]. This PCR reaction uses specific primers based on serovar-specific variations on the capsule loci, capable of discriminating 14 of the 15 recognized *G. parasuis* serovars but not serovars 5 and 12. A new *G. parasuis* serovar-specific PCR scheme containing primers that differentiate serovars 5 and 12 was subsequently described [19]. However, the primer for serovar 12 is based on a hypothetical gene, and further investigations are warranted.

In parallel to molecular serotyping, novel PCR methods were developed to track virulence markers for *G. parasuis* isolates. A group of specific virulence-associated genes, named virulence-associated trimeric autotransporters (*vtaA*) group 1-translocator, were associated with virulent *G. parasuis* strains [20]. Additionally, two different leader sequences were detected in the *vtaA* genes. A test for predicting the virulence potential of *G. parasuis* strains was developed based on the leader sequence of the *vtaA* genes, called LS-PCR [21].

Such epidemiological investigations are essential for designing preventive health programs, supporting vaccine candidate selection, improving pig flow management, and limiting the reliance on prophylactic antimicrobials. This study’s objective was to characterize strains of *G. parasuis* from top pork-producing countries in terms of the relative geographic prevalence of molecular serovars and their relationship with *vtaA* markers.

## Materials and methods

### Isolate collection

**Figure 1:**
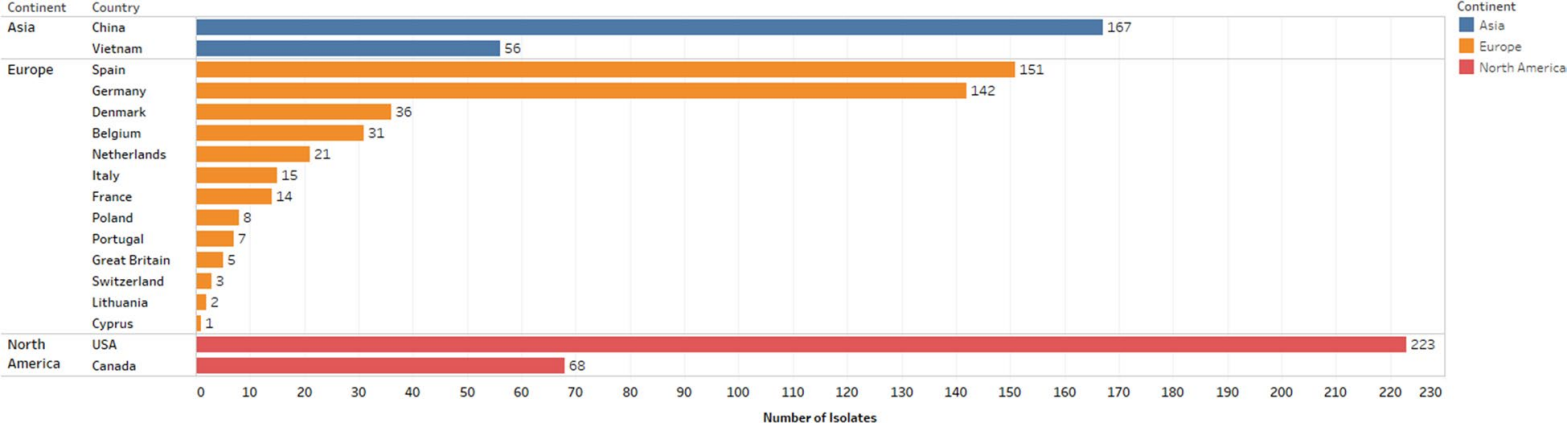
**Distribution of** ***Glaesserella parasuis*** **isolates per country**.

The 950 *G. parasuis* isolates in this collection originated from pigs from different geographical regions; USA (*n* = 223 from 15 different states), Europe (*n* = 436 from 13 different Member States), China (*n* = 167 from 12 different provinces), Vietnam (*n* = 56, from two provinces), and Canada (*n* = 68, from Quebec) (Figure 1, Additional file 1). The dates of collection of isolates ranged between 2014–2018 for USA isolates, 2000–2006, 2015–2016 or unknown for European isolates, 1999–2007 or unknown for Chinese isolates, 2017 for Vietnamese isolates, and 2017–2019 for Canadian isolates (Additional file 1). The *G. parasuis* isolates used in this study came from diagnostic and research laboratories that are national leaders in their own countries regarding swine diseases, including Glässer’s disease. Only isolates with a designated serovar were included. However, more specific information regarding the clinical history and epidemiology was not available for most isolates and was not included in this study.

Data for US isolates (*n* = 223) were obtained from the Iowa State University Veterinary Diagnostic Laboratory (ISU VDL), where isolates were confirmed as *G. parasuis* using Bruker matrix-assisted laser desorption time-of-flight mass spectrometry (MALDI_TOF MS). Data for Canadian strains (*n* = 66) were obtained from the Diagnostic Laboratory of the Faculty of Veterinary Medicine of the University of Montreal (DLFVMUM), where isolates were also firstly identified by MALDI_TOF MS and confirmed by PCR [22]. Data for European isolates were obtained from Spain (IRTA-CReSA, *n* = 275) and Germany (IVD Innovative Veterinary Diagnostics (IVD GmbH), (*n* = 165) and represented 13 different member states. Confirmation of *G. parasuis* by both laboratories was achieved by PCR [22]. Data for Chinese isolates (*n* = 167) and Vietnamese isolates (*n* = 56) were obtained from Huazhong Agricultural University Veterinary Diagnostic Laboratory. The Chinese and Vietnamese isolates were firstly identified with biochemical tests [23] and confirmed by 16S rRNA PCR [25].

Metadata for a subset of these isolates included isolation site, year of isolation, and country of origin (Additional file 1). Metadata for the strains were used to classify isolates considering clinical (internal organs) and non-clinical (URT) source when such information was available. Further classification was performed considering clinical (internal) isolates that were obtained from systemic sites or pulmonary (lungs) and non-clinical (URT) origins. *G. parasuis* strains isolated from internal organs other than the lungs, such as pleura, pericardium, peritoneum, joint, and meninges, were considered systemic strains. All URT strains in this study were isolated from nasal cavities.

### Serotyping PCR

Serotyping PCR was undertaken by each participating laboratory with some laboratory-specific amendments. DNA extraction was achieved by standard methods; typically, a loopful of bacterial colonies were removed from growth media and suspended in 300 µL of 0.01 M sterile phosphate-buffered saline (pH 7.4) without calcium and magnesium (GIBCO/Life Technologies Corporation, Carlsbad, CA, USA) and vortexed. Bacterial DNA was extracted with the MagMAX™ Viral DNA/RNA Isolation Kit (Life Technologies, Carlsbad, CA, USA) using extraction protocol per manufacturer instructions.

All participating laboratories conducted the serotyping PCR as previously described [17] with some (ISU VDL, DLFVMUM, and IVD) using the modifications made by Lacouture et al. [18].

Briefly, each PCR reaction (one for each of the primer mixtures) consisted of 12.5 µL of 2 × Multiplex PCR Master Mix (Qiagen, Hilden, Germany), 2.5 µL of the 100 nmol 1^–1^ of primer mix 1, 2 or 3, 8 µL of nuclease-free water, and 2 µL of extracted DNA. The reaction was run using the following conditions: first cycle at 95 °C for 15 min, followed by 40 cycles of 94 °C for 30 s, 58 °C for 90 s, and 72 °C for 90 s. This was followed by one cycle at 72 °C for 10 min. Electrophoresis and visualization of the PCR products were performed using QIAxcel Capillary Electrophoresis with screen gel software (Qiagen).

For isolates obtained by IRTA-CReSA, PCR conditions were amended using the same primers as Howell et al. [17], but these were tested individually as follows: 94 °C for 5 min, followed by 30 cycles of 94 °C for 30 s, 55 °C for 30 s, and 72 °C for 60 s, and final extension of 72 °C for 7 min. PCR products were visualized by agarose gel electrophoresis.

### Virulence gene PCR (LS-PCR)

Isolates from North America and Europe were tested using a PCR to detect the leader sequence (LS) of the *vtaA* genes domain as a diagnostic tool to predict *G. parasuis* virulence [21]. Briefly, the PCR reaction consisted of 12.5 µL of 2 × Multiplex PCR Master Mix (Qiagen), 1 µL of the 100 nmol 1^–1^ of each primer, 4.5 µL of nuclease-free water, and 2 µL of extracted DNA in a final volume of 25 μL. Cycling conditions were 15 min at 95 °C, followed by 30 cycles of 45 s at 94 °C, 45 s at 52 °C and 1 min at 72 °C, then a final incubation at 72 °C for 10 min.

### Statistical analysis

Associations between serovars, sample site, country of isolation, and LS-PCR results were calculated using Chi-square and Fisher’s exact tests. A multinomial logistic regression model was fit to compare the sample site and serovar classification to the reference (untypeable or NT) serovar. All tests were 2-sided, and a *p*-value of lower than 0.05 was defined as indicating a significant difference. All analyses were completed using SAS version 9.4.

## Results

### Distribution of ***G. parasuis*** serovars

**Figure 2:**
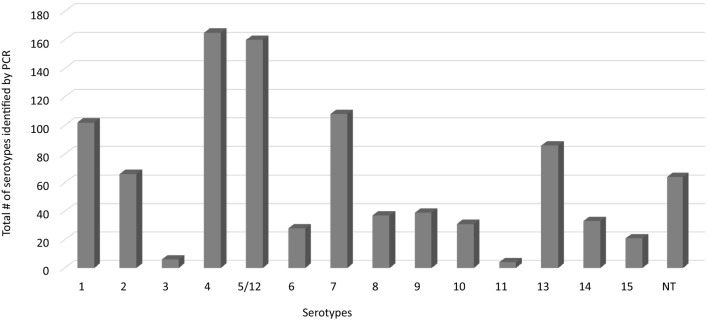
**Frequency distribution of** ***Glaesserella parasuis*** **serovars identified by PCR for all of the isolates in the present study**.

Most of the *G. parasuis* isolates (886/950, 93.3%), were typeable by serotyping PCR, and the remaining 64 (9.7%) were identified as non-typeable (NT). All 15 serovars were identified. Serovars 4 (17.4%), 5/12 (16.8%), 7 (11.4%), 1 (10.7%), and 13 (9.1%) were the most commonly detected serovars across the participating regions (Figure 2, Table 1, Additional files 2, 3, 4).

**Table 1:** **Number and percentage of** ***Glaesserella parasuis*** **serovars per country or region**.

Serovar	USA/%	Canada/%	Europe/%	China/%	Vietnam/%	Total^¶^
1	32/14.3	4/5.9	53/12.2	12/7.2	1/1.8	102
2	28/12.6	4/5.9	13/3	8/4.8	13/23.2	66
3	0/0	1/1.5	0/0	5/3	0/0	6
4	53/23.8	8/11.8	75/17.2	19/11.4	10/17.9	165
5/12	28/12.6	21/30.9	60/13.8	36/21.6	15/26.8	160
6	2/0.9	1/1.5	12/2.8	12/7.2	1/1.8	28
7	41/18.4	11/16.2	51/11.7	4/2.4	1/1.8	108
8	0/0	2/2.9	21/4.8	13/7.8	1/1.8	37
9	0/0	0/0	26/6	7/4.2	6/10.7	39
10	0/0	0/0	18/4.1	6/3.6	7/12.5	31
11	0/0	0/0	3/0.7	1/0.6	0/0	4
13	23/10.3	6/8.8	51/11.7	6/3.6	0/0	86
14	12/5.4	2/2.9	11/2.5	8/4.8	0/0	33
15	1/0.4	1/1.5	18/4.1	1/0.6	0/0	21
NT	3/1.3	7/10.3	24/5.5	29/17.4	1/1.8	64
Total^§^	223/100	68/100	436/100	167/100	56/100	950

^¶^Sum of number of serovars in each row

^§^Sum of number of serovars in each column

### Internal isolates

Information on the site of origin for each isolate was available for 834 of the total 950 *G. parasuis* isolates, allowing classification of *G. parasuis* strains as either nasal or internal (Table 2). Internal isolates represented 81.1% (676/834) of all isolates for which an attributable sample site was recorded (Table 2). The most frequently identified serovars from internal locations included serovars 4, 5/12, 1, 7, and 13 in descending order, with serovars 4 and 5/12 being similarly represented, at 19.2 and 18.2%, respectively.

**Table 2:** **Number and percentage of** ***Glaesserella parasuis*** **serovars per site of isolation**.

Serovar	Nasal/%	Internal/%	Pulmonary*/%	Systemic*/%	Total^¶^
1	19/12	75/11.1	37/12.1	29/13.7	94
2	6/3.8	53/7.8	31/10.1	14/6.6	59
3	0/0	6/0.9	0/0	1/0.5	6
4	18/11.4	130/19.2	83/27	29/13.7	148
5/12	13/8.2	124/18.3	45/14.7	47/22.2	137
6	7/4.4	19/2.8	2/0.7	5/2.4	26
7	8/5.1	74/10.9	37/12.1	33/15.6	82
8	19/12	16/2.4	3/1	1/0.5	35
9	18/11.4	17/2.5	6/2	4/1.9	35
10	15/9.5	14/2.1	7/2.3	1/0.5	29
11	0/0	4/0.6	2/0.7	1/0.5	4
13	8/5.1	63/9.3	28/9.1	30/14.2	71
14	1/0.6	30/4.4	15/4.9	7/3.3	31
15	17/10.8	2/0.3	0/0	1/0.5	19
NT	9/5.7	49/7.2	11/3.6	9/4.2	58
Total^§^	158/100	676/100	307/100	212/100	834

*Information was available for 519 internal isolates, which were divided into pulmonary and systemic

^¶^Sum of nasal and internal isolates in each row

^§^Sum of values in each column

Further classification of internal isolates into pulmonary and systemic was possible for a subset of 519 of these isolates. Almost sixty percent of these further classified isolates (307/519, 59.2%) were obtained from pulmonary sites, while 40.8% (212/519) were of systemic origin (Table 2). Serovar 4 strains were more frequently isolated from lung samples than serovar 5/12 strains (27 and 14.7%, respectively). In contrast, when considering systemic isolates separately, the percentage of serovar 5/12 strains was higher compared with serovar 4 (22.2 and 13.7%, respectively) (Table 2). Likewise, both serovars 7 and 13 were more frequently detected within internal samples, than nasal samples. However, while serovar 7 was similarly represented within both pulmonary and systemic collections (12.1 and 15.6% of each collection, respectively), the percentage of serovar 13 strains was higher in the systemic collection compared with the pulmonary one (14.2 and 9.1%, respectively) (Table 2).

Clinically relevant serovars from internal sites, such as serovars 4, 5/12, 7, 1, and 13, were among the six serovars more frequently observed in North America and Europe, with similar distribution among systemic and pulmonary sites (Tables 3, 4, 5). Among Asian isolates, serovar 5/12 was the most prevalent, followed by serovars 4 and 2 (Table 3).

**Table 3:** **Number and percentage of Internal** ***Glaesserella parasuis*** **serovars per country or region**.

Serovar	USA/%	Canada/%	Europe/%	China/%	Vietnam/%	Total¶
1	31/14.8	3/6.7	32/13.7	9/5.7	0/0	75
2	27/12.9	3/6.7	8/3.4	8/5.1	7/22.6	53
3	0/0	1/2.2	0/0	5/3.2	0/0	6
4	51/24.3	1/2.2	54/23.2	18/11.5	6/19.4	130
5/12	28/13.3	15/33.3	35/15	32/20.4	14/45.2	124
6	2/1	1/2.2	4/1.7	12/7.6	0/0	19
7	34/16.2	8/17.8	28/12	4/2.5	0/0	74
8	0 /	1/2.2	3/1.3	12/7.6	0/0	16
9	0/0	0/0	10/4.3	7/4.5	0/0	17
10	0/0	0/0	4/1.7	6/3.8	4/42.9	14
11	0/0	0/0	3/1.3	1/0.6	0/0	4
13	22/10.5	4/8.9	32/13.7	5/3.2	0/0	63
14	12/5.7	1/2.2	9/3.9	8/5.1	0/0	30
15	0/0	1/2.2	0/0	1/0.6	0/0	2
NT	3/1.4	6/13.3	11/4.7	29/18.5	0/0	49
Total^§^	210/100	45/100	233/100	157/100	31/100	676

^¶^Sum of number of serovars in each row.

^§^Sum of number of serovars in each column.

**Table 4:** **Number and percentage of Pulmonary  Glaesserella parasuis serovars per country or region**.

Serovar	USA/%	Europe/%	Vietnam/%	Total^¶^
1	15/11.9	22/14.7	0/0	37
2	17/13.5	7/4.7	7/22.6	31
4	39/31	38/25.3	6/19.4	83
5/12	15/11.9	16/10.7	14/45.2	45
6	1/0.8	1/0.7	0/0	2
7	19/15.1	18/12	0/0	37
8	0/0	3/2	0/0	3
9	0/0	6/4	0/0	6
10	0/0	3/2	4/12.9	7
11	0/0	2/1.3	0/0	2
13	7/5.6	21/14	0/0	28
14	11/8.7	4/2.7	0/0	15
NT	2/1.6	9/6	0/0	11
Total^§^	126/100	150/100	31/100	307

^¶^Sum of number of serovars in each row.

^§^Sum of number of serovars in each column.

There is no information whether Canadian and Chinese internal isolates originated from pulmonary sites.

**Table 5:** **Number and percentage of Systemic** ***Glaesserella parasuis*** **serovars per country or region**.

Serovar	USA/%	Canada/%	Europe/%	Total^¶^
1	16/19	3/6.7	10/12	29
2	10/11.9	3/6.7	1/1.2	14
3	0/0	1/2.2	0/0	1
4	12/14.3	1/2.2	16/19.3	29
5/12	13/15.5	15/33.3	19/22.9	47
6	1/1.2	1/2.2	3/3.6	5
7	15/17.9	8/17.8	10/12	33
8	0/0	1/2.2	0/0	1
9	0/0	0/0	4/4.8	4
10	0/0	0/0	1/1.2	1
11	0/0	0/0	1/1.2	1
13	15/17.9	4/8.9	11/13.3	30
14	1/1.2	1/2.2	5/6	7
15	0/0	1/2.2	0/0	1
NT	1/1.2	6/13.3	2/2.4	9
Total^§^	84/100	45/100	83/100	212

^¶^Sum of number of serovars in each row.

^§^Sum of number of serovars in each column.

There is no information whether Asian internal isolates originated from systemic sites.

The highest proportion of untypable internal isolates (18.5%, 29/157) was found among Chinese isolates, while only 1.4% (3/210) of USA internal isolates were untypable (Table 3).

### Nasal isolates

Nasal isolates represented 18.1% (158/834) of all isolates for which an attributable sample site was recorded and belonged to a variety of serovars, especially serovars 8 (12%), 1 (12%), 4 (11.4%), 9 (11.4%), 15 (10.8%) and 10 (9.5%) (Table 2). While serovars 1 and 4 were more consistently found within systemic or pulmonary sites, serovars 8, 9, 15, and 10 were predominantly obtained from nasal samples (Table 2).

Most of the nasal isolates in this study were obtained from Europe (76.6%, 121/158) (Table 6). The most prevalent nasal serovars in Europe were 8, 15, 1, 4, 9, and 10. Most of these serovars were not prevalent among internal samples, except for serovars 1 and 4, which were also prevalent among pulmonary and systemic sites (Tables 3, 4, 5, 6).

**Table 6:** **Number and percentage of Nasal** ***Glaesserella parasuis*** **serovars per country or region**.

Serovar	USA/%	Europe/%	China/%	Vietnam/%	Total^¶^
1	1/50	14/11.6	3/30	1/4	19
2	0/0	0/0	0/0	6/24	6
4	1/50	12/9.9	1/10	4/16	18
5/12	0/0	8/6.6	4/40	1/4	13
6	0/0	6/5	0/0	1/4	7
7	0/0	7/5.8	0/0	1/4	8
8	0/0	17/14	1/10	1/4	19
9	0/0	12/9.9	0/0	6/24	18
10	0/0	12/9.9	0/0	3/12	15
13	0/0	7/5.8	1/10	0/0	8
14	0/0	1/0.8	0/0	0/0	1
15	0/0	17/14	0/0	0/0	17
NT	0/0	8/6.6	0/0	1/4	9
Total^§^	2/100	121/100	10/100	25/100	158

^¶^Sum of number of serovars in each row.

^§^Sum of number of serovars in each column.

There were no nasal *G. parasuis* isolates submitted from Canada.

Besides these European nasal isolates, 35 *G. parasuis* nasal isolates were from Asian countries, including 25 from Vietnam and ten from China. Most of the nasal isolates from China were either from serovar 5/12 or 1, while most of the nasal isolates from Vietnam were from serovars 2, 9, 4, and 10 (Tables 6). However, only serovars 1 and 9 were predominantly higher among nasal isolates. The other serovars were also frequently found among internal isolates (Table 3).

### Association between *Glaesserella parasuis* serovars and site of isolation

Results of the chi-square analysis showed an association between sample type (nasal/internal) and serovar (*p* < 0.001). A multinomial logistic regression model was further used to predict serovar classification based on sample type. Given the sample type (nasal/internal), samples collected from the nasal cavity had a higher probability of being of serovars 8, 9, 10, or 15 (*p* < 0.05), but no association was found between serovar and internal samples, during this initial analysis.

Using the same model, internal isolates were classified as either pulmonary or systemic and compared against nasal isolates for the subsequent analysis. Given the sample type, systemic isolates had a higher probability of being of serovar 5/12, 13, or 7 compared to the nasal sample collection (*p* < 0.01). In comparison, pulmonary isolates had a higher likelihood of being serovars 2, 4, 7, or 14 (*p* < 0.01). No statistical significance was observed when comparing systemic and pulmonary isolates. These results agree with Olvera et al. [20], where pulmonary isolates could be either virulent or non-virulent since the lung can be populated by upper respiratory tract commensal microbiota and strains that harbor certain virulence factors that enable them to survive the lung defenses and invade systemically. As shown in Table 2, 14/53 and 7/31 of serovars 2 and 14, respectively, were also found on systemic sites.

### Association between *Glaesserella parasuis* serovars and LS-PCR result

*G. parasuis* isolates were classified as potentially virulent based on the presence or absence of specific leader sequences within their *vtaA* genes, using multiplex LS-PCR. Within this *G. parasuis* collection, 727 isolates were tested by LS-PCR among North American and European isolates (Table 7). Overall, 85.7% (623/727) of the *G. parasuis* isolates were classified as virulent by LS-PCR, 14.3% (104/727) were classified as non-virulent by LS-PCR, and 223 isolates were not tested (Table 7).

**Table 7:** **Number and percentage of virulent and non-virulent isolates within the same serovar**.

Serovar	Virulent by LS-PCR/%	Non-Virulent by LS-PCR/%	Not Tested by LS-PCR/%	Total^¶^
1	89/87.3	0/0	13/12.7	102
2	44/66.7	1/1.5	21/31.8	66
3	0/0	1/16.7	5/83.3	6
4	136/82.4	0/0	29/17.6	165
5/12	106/66.3	3/1.9	51/31.9	160
6	4/14.3	11/39.3	13/46.4	28
7	101/93.5	2/1.9	5/4.6	108
8	0/0	23/62.2	14/37.8	37
9	6/15.4	20/51.3	13/33.3	39
10	2/6.5	16/51.6	13/41.9	31
11	3/75	0/0	1/25	4
13	77/89.5	3/3.5	6/7	86
14	25/75.8	0/0	8/24.2	33
15	3/14.3	17/81	1/4.8	21
NT	27/42.2	7/10.9	30/46.9	64
Total^§^	623/65.6	104/10.9	223/23.5	950

^¶^Sum of number of serovars in each row.

^§^Sum of number of serovars in each column.

Most of the disease-associated isolates, including 1, 2, 4, 5/12, 7, 13, and 14, were considered virulent by LS-PCR, with few exceptions where these virulent serovars were deemed to be non-virulent by LS-PCR (10/578) (Table 7). In contrast, most of the isolates from the nasal cavity, including serovars 6, 9, 10, and 15, were considered non-virulent by LS-PCR (Table 8), with a significant association between serovar and classification as virulent or non-virulent by LS-PCR (*p* < 0.001). Further analysis was performed within each serovar to evaluate the association of virulent and non-virulent strains with the site of isolation. A significant association was found for serovars 5/12 and 7, which had a higher chance of being virulent and from internal sites (systemic or pulmonary). Contrarily, serovar 10 had a higher chance of being non-virulent and from a nasal site (Table 8).

**Table 8:** **Distribution of** ***Glaesserella parasuis*** **serovars by site of isolation and LS-PCR results**.

	LS-PCR	Systemic	Pulmonary	Nasal	*P*-value
Serovar 1	*Virulent*	29	37	15	
	*Non-Virulent*	0	0	0	Not Tested*
Serovar 2	*Virulent*	14	23	0	
	*Non-Virulent*	0	1	0	0.439
Serovar 3	*Virulent*	0	0	0	
	*Non-Virulent*	1	0	0	Not Tested
Serovar 4	*Virulent*	14	23	0	
	*Non-Virulent*	0	1	0	0.439
Serovar 5/12	*Virulent*	47	30	6	
	*Non-Virulent*	0	1	2	0.002
Serovar 6	*Virulent*	1	0	2	
	*Non-Virulent*	4	2	4	0.612
Serovar 7	*Virulent*	33	37	5	
	*Non-Virulent*	0	0	2	0.000
Serovar 8	*Virulent*	0	0	0	
	*Non-Virulent*	1	3	17	Not Tested
Serovar 9	*Virulent*	0	3	1	
	*Non-Virulent*	4	3	11	0.056
Serovar 10	*Virulent*	1	1	0	
	*Non-Virulent*	0	2	12	0.007
Serovar 11	*Virulent*	1	2	0	
	*Non-Virulent*	0	0	0	Not Tested
Serovar 13	*Virulent*	30	26	6	
	*Non-Virulent*	0	2	1	0.188
Serovar 14	*Virulent*	7	15	1	
	*Non-Virulent*	0	0	0	Not Tested
Serovar 15	*Virulent*	0	0	2	
	*Non-Virulent*	1	0	15	0.716

*Not Tested: All isolates from this serovar had the same LS-PCR result.

*P*-value < 0.05 was considered significant.

## Discussion

Serotyping has been a necessary means of characterizing *G. parasuis* isolates. Using a novel serotyping PCR method [17, 18], we described the distribution of *G. parasuis* serovars globally and within a geographic region. The data collected in this study are from different diagnostic laboratories and research centers. Even though they serve as centers of expertise on the diagnosis of swine diseases, the dataset used in this study is limited to isolates available in these laboratories and, therefore, do not represent a true prevalence of *G. parasuis* serovars. Additionally, some of the strains from Europe (57/436) and China (167/223) were isolated more than ten years ago or have an unknown date of isolation. Thus, the actual distribution (prevalence) of *G. parasuis* serovars is still unknown. Even though our data do not represent prevalence, most of these isolates originate from field cases and reflect strains frequently found in disease-associated cases, and thus useful information for swine practitioners and researchers.

The serovars’ overall distribution varied slightly among countries, and four serovars (1, 4, 5/12, and 7) accounted for half of all isolates. Serovars 4 and 5/12, in particular, were consistently represented across all regions in this study and previous studies [13, 22–28].

The serovars of utmost clinical significance were identified in samples from internal sites (serovars 4, 5/12, 1, 7, and 13) in this study. These serovars have been previously considered disease-associated [12, 13, 22–30]. Variable sample sizes limited our ability to discern significant differences in serovar prevalence at the country level. The relative scarcity of serovar 4 isolates among Canada's internal samples was notable, and serovars 1, 7, and 13 appeared relatively similarly scarce among internal samples from China and Vietnam.

An important finding in this present study and other recent studies [28–30] was that serovar 7 is a significant disease-associated serovar of *G. parasuis*. In this study, serovar 7 was assigned to 10.9% of all internal samples (ranked 4^th^ most prevalent serovar) and 15.6% of all systemic isolates (ranked second most prevalent serovar). Serovar 7 was initially found non-virulent by intraperitoneal challenge [12], and it was the most prevalent serovar identified from the URT of healthy pigs in China [14]. Conversely, a serovar 7 field strain was able to cause disease in one snatch-farrowed colostrum-deprived pig when inoculated intranasally and was consistently isolated from pulmonary and systemic locations from pigs affected by Glasser’s disease in Germany [28]. Additionally, the serovar 7 reference strain 174 caused disease in colostrum-deprived and commercial pigs after experimental inoculation [29, 30], demonstrating this serovar’s pathogenic potential [31].

Based on their detection in fewer than 1% of all internal samples, the least clinical significance serovars were serovars 3, 11, and 15. Serovars 3 and 11 were previously considered non-virulent [12]. Still, the apparent limited clinical significance of serovar 15 contradicted previous findings [12], highlighting the limitations of small-scale challenge studies as indicators of virulence. Isolates of serovar 10 have been reported as disease and non-disease associated [31]. Still, while the present study found serovar 10 in 9.9% of nasal samples from Europe (the region supplying the most significant bulk of nasal samples), it was found in only 1.7% of internal samples from Europe – along with a small cluster of internal isolates from China and Vietnam. Thus, the present study indicates only limited significance for this serovar.

Nevertheless, pigs harbor various *G. parasuis* strains in their URT, and potentially pathogenic serovars can be isolated from the URT of healthy pigs [9, 14]. In this study, URT isolates originated mainly from Europe and Asia, representing 16.6% of this study’s collection. Serovars 6, 8, 9, 10, and 15 were amongst the most commonly identified (43.6%) in the URT. In comparison, these serovars represented only 7.2% of all internal isolates. Serovars 10 and 15 have been traditionally considered virulent, even though they are not prevalent as disease-associated serovars worldwide, and were classified as non-virulent by the LS-PCR. Interestingly, serovars 6 and 8 Asian isolates were also frequently isolated from Chinese samples’ internal sites, representing an increase of more virulent strains belonging to these serovars in Asia.

Different pathogenic properties have also been described for *G. parasuis* serovar 4 strains. The reference isolate for serovar 4 was initially isolated from the nose of a healthy pig and considered mildly virulent [12]. In our study, serovar 4 isolates were mainly associated with pulmonary sites (83/130, 64%), followed by systemic (29/130) and nasal sites (18/130). In an earlier study, North American isolates of serovar 4 were mainly isolated from lung samples of pigs with pneumonia and other systemic sites, but not from URT samples [22]. When two serovar 4 European field isolates were used to infect pigs experimentally, one could reproduce Glasser’s disease, while the other could not [31]. These results are consistent with other reports showing serovar 4 isolates clustering with virulent and non-virulent genotypes [31–33]. These findings illustrate how isolates of the same serovar can have different degrees of virulence. Additionally, some *G. parasuis* serovars’ virulence may have been wrongly assigned by the Kielstein and Rapp-Gabrielson scheme [12].

Glasser’s disease is a complex multifactorial disease, and several different putative virulence genes have been identified [34–37]. Still, little experimental data is available to correlate these virulence genes with clinical disease. However, empirical evidence exists, demonstrating the group 1 *vtaA* genes’ role in *G. parasuis* virulence [21, 38, 39]. Therefore, to help identify virulent *G. parasuis* strains, a new PCR, based on the leader sequence of the *vtaA* genes, was developed [21].

In the present study, most internal isolates were identified as virulent by LS-PCR, while most nasal isolates were non-virulent. Our results agree with previous reports [25, 28, 39–41] and supported our disease association observations for serovar 7 isolates. In our study, most serovar 7 (75/77, 97.4%) strains were internal and classified as virulent by the LS-PCR. Only two serovar 7 LS-PCR negatives were found in the collection, and these were from nasal samples. In vitro analysis was not performed in this study to further characterize the virulence of the strains. Previous genome analysis performed on a serovar 7 reference strain classified as virulent by LS-PCR, but susceptible to both phagocytosis and serum demonstrated a reduction in the number of *vtaAs* in that strain, which may represent a strain with an intermediate phenotype [21].

Nevertheless, some discordant results were also found within the LS-PCR data. We found that a small number of isolates from typically non-disease-associated serovars (such as 6, 8, and 9), and classified as non-virulent by LS-PCR, were recovered from systemic sites. In this case, other environmental factors or coinfections could be responsible for creating the necessary conditions for less virulent strains to invade systemically [21, 31, 40]. Even though *vtaA* genes are positively correlated with *G. parasuis* virulent strains and can be used as a predictor of virulence, other factors may also be necessary for disease development [25, 32, 40].

In conclusion, the overall distribution of *G. parasuis* serovars remains predominantly constant over the past 20 years, with few serovars representing most of the strains isolated from Glasser’s disease-affected pigs and noting the confirmation of serovar 7 as a globally relevant serovar. Even though the serovar and virulence relationship is not definitive, serotyping is still suitable for the epidemiological characterization of *G. parasuis* in pig farms, helping implement more adequate control measures, such as selecting isolates to be included in vaccines. When serotyping is not available, the site of isolation can become crucial for screening disease-associated isolates since systemic strains are likely the ones that possess the necessary virulent mechanisms for disease development. To date, *vtaA* genes are the most well-characterized *G. parasuis* virulence factors. This survey of global *G. parasuis* isolates confirms that the LS-PCR is useful for *G. parasuis* virulence prediction.

## Supplementary Information

**Additional file 1. Metadata of Glaesserella parasuis isolates including isolation site, year of isolation, and country of origin.**

**Additional file 2. Distribution of Glaesserella parasuis serovars in North America.**

**Additional file 3. Distribution of Glaesserella parasuis serovars in Europe.**

**Additional file 4. Distribution of Glaesserella parasuis serovars in Asia.**

## Data Availability

All data generated or analyzed during this study are included in this published article and its supplementary information files.
