# Supplementary figures and images for: Molecular characterization of Glaesserella parasuis strains isolated from North America, Europe and Asia by serotyping PCR and LS-PCR
Source: Vet Res. 2021 May 12;52:68. doi: 10.1186/s13567-021-00935-9 (PMC8117636; doi:10.1186/s13567-021-00935-9)

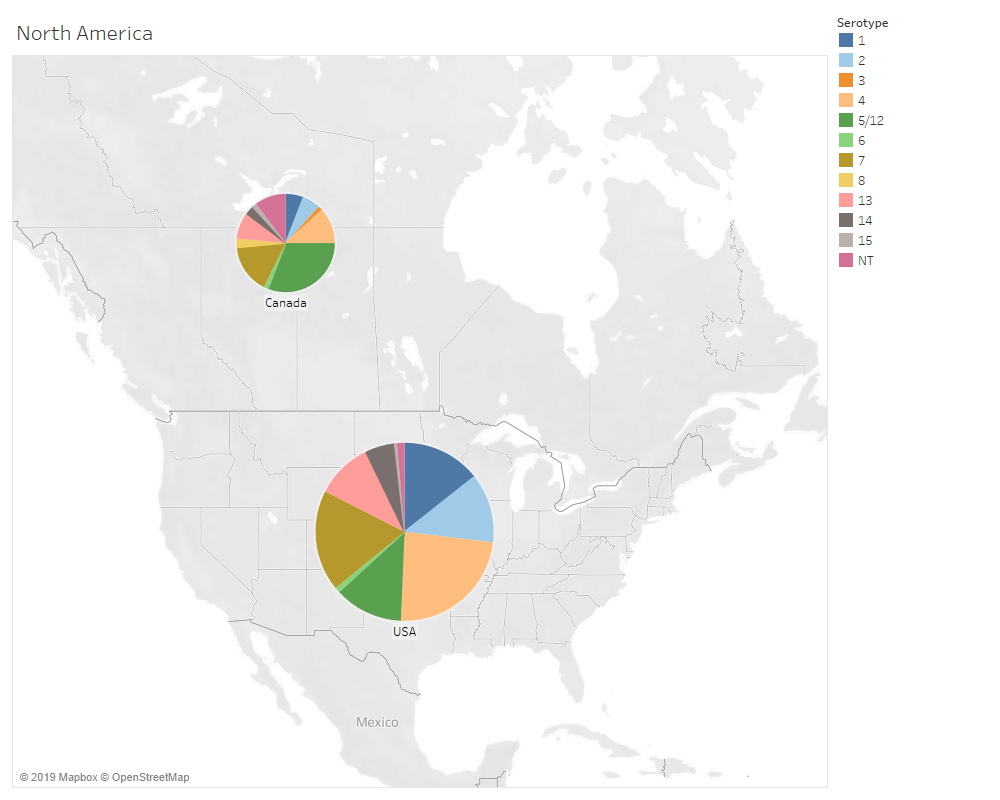

Supplement: Supplementary file 2 — Additional file 2. Distribution of Glaesserella parasuis serovars in North America. [file 13567_2021_935_MOESM2_ESM.docx]

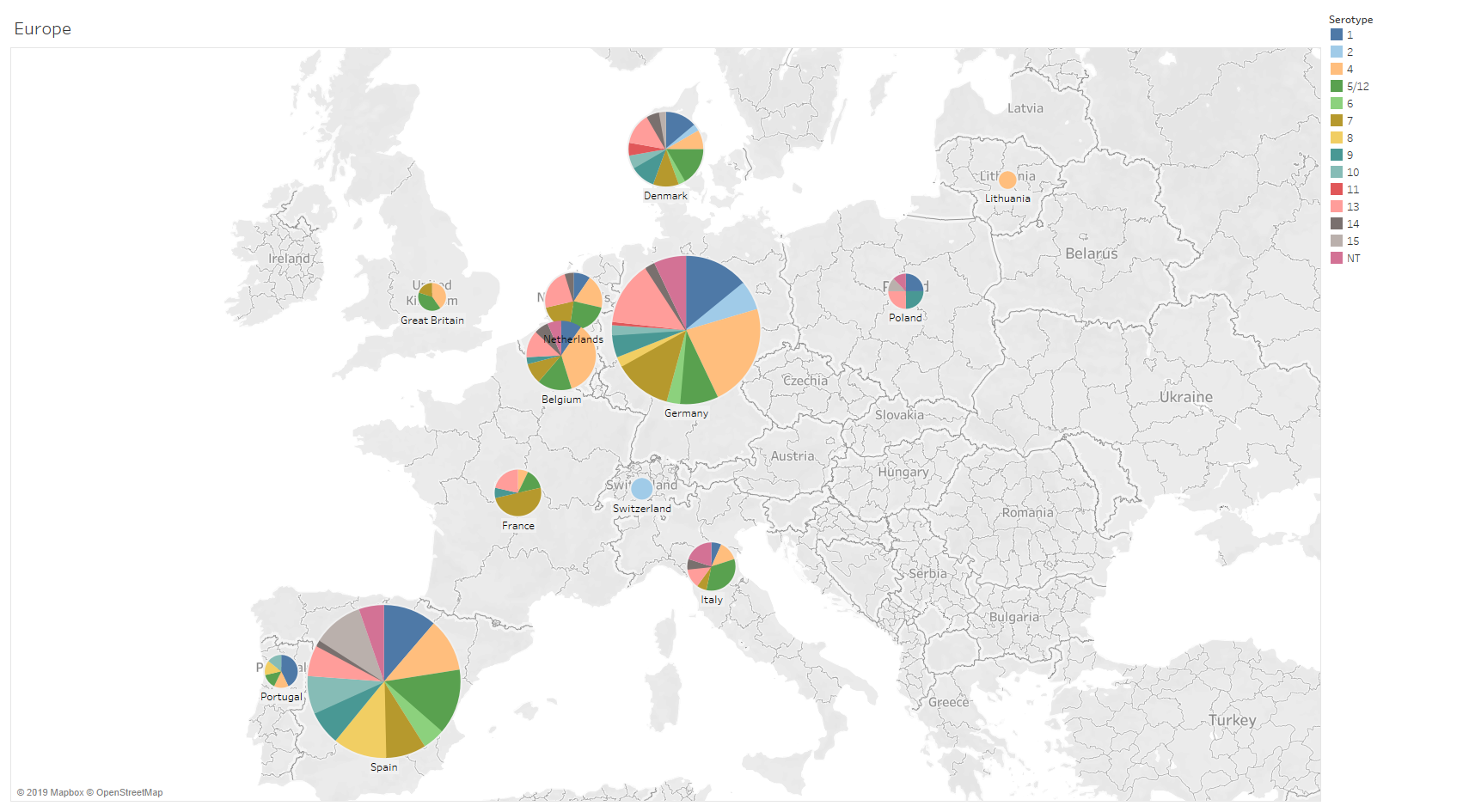

Supplement: Supplementary file 3 — Additional file 3. Distribution of Glaesserella parasuis serovars in Europe. [file 13567_2021_935_MOESM3_ESM.docx]

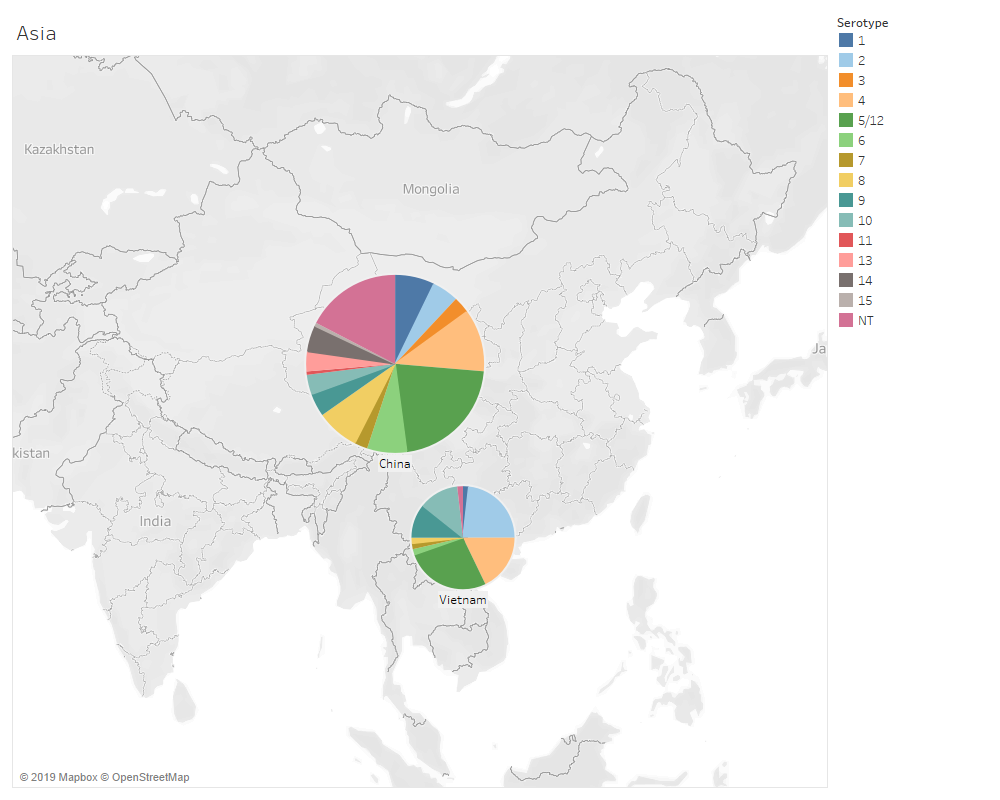

Supplement: Supplementary file 4 — Additional file 4. Distribution of Glaesserella parasuis serovars in Asia. [file 13567_2021_935_MOESM4_ESM.docx]
